# Gene Clusters Located on Two Large Plasmids Determine Spore Crystal Association (SCA) in *Bacillus thuringiensis* Subsp. *finitimus* Strain YBT-020

**DOI:** 10.1371/journal.pone.0027164

**Published:** 2011-11-04

**Authors:** Yiguang Zhu, Fang Ji, Hui Shang, Qian Zhu, Pengxia Wang, Chengchen Xu, Yun Deng, Donghai Peng, Lifang Ruan, Ming Sun

**Affiliations:** 1 State Key Laboratory of Agricultural Microbiology, College of Life Science and Technology, Huazhong Agricultural University, Beijing, People's Republic of China; 2 School of Medicine, Jianghan University, Wuhan, People's Republic of China; Loyola University Medical Center, United States of America

## Abstract

Crystals in *Bacillus thuringiensis* are usually formed in the mother cell compartment during sporulation and are separated from the spores after mother cell lysis. In a few strains, crystals are produced inside the exosporium and are associated with the spores after sporulation. This special phenotype, named ‘spore crystal association’ (SCA), typically occurs in *B. thuringiensis* subsp. *finitimus*. Our aim was to identify genes determining the SCA phenotype in *B. thuringiensis* subsp. *finitimus* strain YBT-020. Plasmid conjugation experiments indicated that the SCA phenotype in this strain was tightly linked with two large plasmids (pBMB26 and pBMB28). A shuttle bacterial artificial chromosome (BAC) library of strain YBT-020 was constructed. Six fragments from BAC clones were screened from this library and discovered to cover the full length of pBMB26; four others were found to cover pBMB28. Using fragment complementation testing, two fragments, each of approximately 35 kb and located on pBMB26 and pBMB28, were observed to recover the SCA phenotype in an acrystalliferous mutant, *B. thuringiensis* strain BMB171. Furthermore, deletion analysis indicated that the crystal protein gene *cry26Aa* from pBMB26, along with five genes from pBMB28, were indispensable to the SCA phenotype. Gene disruption and frame-shift mutation analyses revealed that two of the five genes from pBMB28, which showed low similarity to crystal proteins, determined the location of crystals inside the exosporium. Gene disruption revealed that the three remaining genes, similar to spore germination genes, contributed to the stability of the SCA phenotype in strain YBT-020. Our results thus identified the genes determining the SCA phenotype in *B. thuringiensis* subsp. *finitimus*.

## Introduction

*Bacillus thuringiensis* is a Gram-positive, spore-forming bacterium with one of its most important features being the formation of parasporal crystals. The insecticidal activity of *B. thuringiensis* lies primarily in its parasporal crystals. *B. thuringiensis* strains carry one or more different crystal genes which are usually located on large, transmissible plasmids [1]. Crystal protein is typically deposited against the forespore and develops outside the exosporium. Parasporal crystals are then separated from spores after lysis of the mother cell. However, in a few strains, such as in *B. thuringiensis* subsp. *finitimus* strains [2], [3] and *B. thuringiensis* subsp. *oyamensis* strain LBIT-113 [4], the parasporal crystals are located between the exosporium and the spore coat and continue to adhere to the spore after mother cell lysis. This phenotype has been previously described as spore-crystal association (SCA) [5].

The SCA phenotype was identified a half century ago [6]. SCA strains were originally designated as *Bacillus finitimus*; “finitimus” meaning “neighboring” or “adjacent” in Latin, and this species was later made a subspecies of *B. thuringiensis* [7]. Debro *et al.* [2] described that the SCA phenotype depended on a 98 MDa plasmid, suggesting that the plasmid contained all the genes essential for crystal formation within the exosporium, and that the inclusion that formed within the exosporium contained a major polypeptide of approximately 135 kDa. Two crystal protein encoding genes, *cry26Aa1* and *cry28Aa1*, were identified from *B. thuringiensis* subsp. *finitimus* B-1166 VKPM; encoding 131 and 125 kDa proteins respectively [3]. An unusual nontoxic strain of *B. thuringiensis* subsp. *oyamensis* was isolated from living larvae of *Anopheles pseudopunctipennis* [4]. In this strain, the crystal protein was found to be enclosed within the exosporium and composed of two proteins of 88 and 54 kDa. A survey of *B. thuringiensis* strains isolated from Spanish citrus orchards, conducted by Vidal-Quist *et al.* [8], showed that 25 out of 376 strains produced crystals that adhered to spores. Four morphological types of crystals with four different protein profiles were described using SDS-PAGE.

To date, the gene(s) conferring such localization have not been reported. *B. thuringiensis* subsp. *finitimus* strain YBT-020 is a typical strain with the SCA phenotype. In our previous studies, two crystal protein genes, *cry26Aa* and *cry28Aa*, were isolated from this strain. When *cry26Aa* and *cry28Aa* were transferred into the acrystalliferous *B. thuringiensis* strain BMB171, the crystals were separated from the spores after mother cell lysis, even when they were transferred into the plasmid-cured strain of YBT-020, that had been cured of all plasmids. These results revealed that the expression of *cry26Aa* and *cry28Aa* alone from their own promoters was not sufficient for SCA phenotype [5].

To isolate the key genes that determining the SCA phenotype in strain YBT-020, the following work was performed: testing which plasmid was required for the SCA phenotype by plasmid conjugation, and then constructing a shuttle bacterial artificial chromosome (BAC) library for complementation testing to enable screening of clones exhibiting the SCA phenotype. We found that two native, large plasmids, pBMB26 and pBMB28, were essential for the formation of SCA phenotype, and two 35 kb fragments located on plasmids pBMB26 and pBMB28 were able to recover the SCA phenotype in an acrystalliferous mutant strain BMB171. Deletion analysis and gene disruption indicated that six genes are indispensable for the SCA phenotype in strain YBT-020.

## Results

### Discovery of native plasmids pBMB26 and pBMB28 determining the SCA phenotype

**Figure 1:**
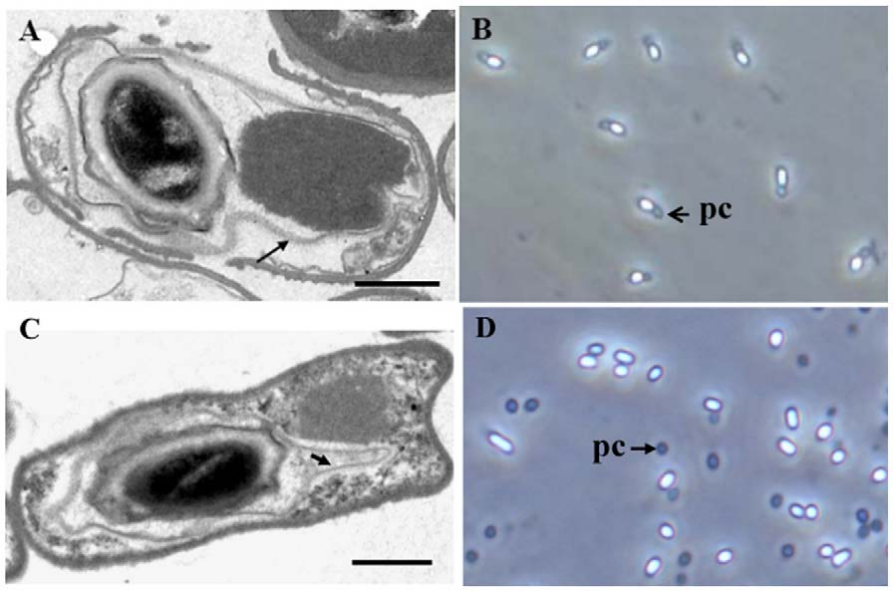
Micrographs of parasporal crystals from transconjugants. (A) Electron micrograph of a thin section of transconjugant with the SCA phenotype during sporulation. (B) Transconjugants with the SCA phenotype after sporulation; (C) Electron micrograph of a thin section of transconjugant with separated crystal during sporulation. (D) Transconjugants with separated crystal after sporulation. (A) and (C) were Grown for 20 h, Scale bar = 0.5 µm; the arrowheads indicate the exosporium. (B) and (D) were grown for 48 h and observed by phase-contrast microscopy. pc = parasporal crystal. Magnification, ×1,000.

Strain YBT-020 harbors two native plasmids, named pBMB26 and pBMB28. In our previous study [9], a plasmid-curing experiment suggested that plasmid pBMB26, harboring the crystal protein gene *cry26Aa*, was indispensable for the SCA phenotype in strain YBT-020. To test whether plasmid pBMB26 contains all the genes essential for the SCA phenotype, a plasmid conjugation experiment was performed. The conjugational donor, strain BMBJ1, was generated by inserting a chloramphenicol resistance cassette into gene *cry26Aa* via homologous recombination. By screening of the acrystalliferous mutant strain BMB171, a spontaneous rifampin resistance mutant was obtained. Plasmid pBMB0617, harboring gene *cry26Aa*, was transformed into this strain to generate the recipient strain BMB171R1. Two kinds of phenotype were isolated from the transconjugants, with crystals enclosed inside spores (Fig. 1A, 1B), or crystals separated from spores (Fig. 1C, 1D). The morphology of fifty randomly selected transconjugants was observed, and the ratio of the two phenotypes was found to be approximately 1∶4.

Based on the complete sequences of plasmids pBMB26 and pBMB28 [10], PCR primers were designed to detect the existence of both plasmids. PCR verification showed that the strains with separated crystals contained plasmid pBMB26, while the strains with the SCA phenotype contained the two plasmids. This mating experiment suggested that the two plasmids together determined the SCA phenotype.

### Characterization of fragments from pBMB26 and pBMB28 recovering the SCA phenotype in BMB171

**Figure 2:**
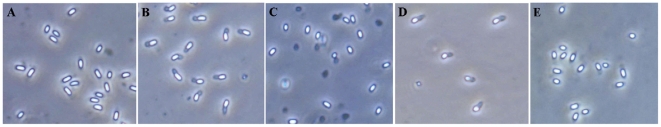
Phase-contrast micrographs of recombinant strains after growth for 48 h. (A) BMB1151 (pBMB26-cured mutant of YBT-020). (B) Strain BMB1151 containing the 35 kb fragment of pBMB26. (C) Strain BMB171 containing the 35 kb fragment of pBMB26. (D) Strain BMB171 containing the fragments of pBMB26 and pBMB28. (E) Strain BMB171 containing the 35 kb fragment of pBMB28. Magnification, ×1,000.

To locate the critical regions detemining the SCA phenotype, six fragments covering the full length of pBMB26, and four others covering pBMB28, were screened from the shuttle BAC library of strain YBT-020. The plasmid pBMB26-cured mutant BMB1151 of strain YBT-020 [10], in which crystals were not formed (Fig. 2A), was used as a host strain to locate the crucial region for the SCA phenotype within plasmid pBMB26. Six BAC clones, covering different regions of pBMB26, were transferred into mutants BMB1151 and BMB171 by electroporation. The SCA phenotype of the transformants was detected by microscopic observation after sporulation. A fragment of 35 kb (pBMB275), carrying *cry26Aa*, was confirmed to direct the formation of crystals adhering to spores, and to recover the SCA phenotype in the pBMB26-cured mutant BMB1151 of strain YBT-020 (Fig. 2B), but not in acrystalliferous mutant BMB171 (Fig. 2C). This further suggested that both pBMB26 and pBMB28 were indispensable to the SCA phenotype.

Following this, we determined the location of the crucial region within plasmid pBMB28 that was essential to the SCA phenotype. As a requirement of resistance screening, the 35 kb fragment (pBMB275) was inserted into another shuttle BAC vector pEMB0603, to give rise to pBMB275A. Using the same methods, four BAC clones, covereing different regions of pBMB28, were transferred into strains BMB171/pBMB275A and BMB171. The SCA phenotype of the transformants was detected after sporulation. A 35 kb fragment (pBMB251) was demonstrated to recover the SCA phenomenon in strain BMB171/pBMB275A (Fig. 2D), but not in strain BMB171 (Fig. 2E). Thus, by large fragment complementation testing, two 35 kb fragments, from plasmids pBMB26 and pBMB28, were identified to determine the SCA phenotype.

### Determination of minimal regions essential to the SCA phenotype

**Figure 3:**
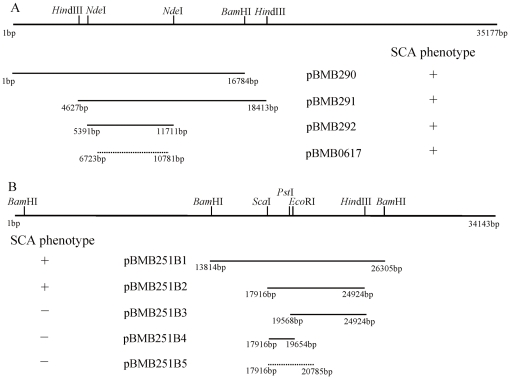
The search for minimal DNA fragments essential to the SCA phenotype in *B. thuringiensis* strain BMB171. (A) Determination of the minimal sequence of pBMB275 (a fragment of pBMB26) in strain BMB171 harboring pBMB251A (a fragment of pBMB28). (B) Determination of the minimal sequence of pBMB251 (a fragment of pBMB28) in strain BMB171 harboring pBMB275A (a fragment of pBMB26). The ability of the region to recover SCA is indicated by “+”, and inability by “−”.

**Figure 4:**
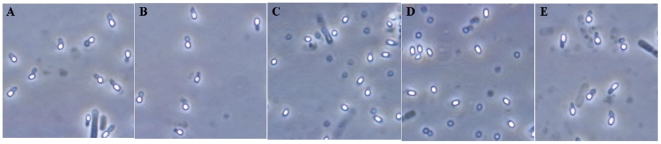
Phase-contrast micrographs of different plasmid derivatives of pBMB275 and pBMB251 in strain BMB171 after growth for 48 h. (A) Strain BMB171/pBMB251A+pBMB0617. (B) Strain BMB171/pBMB275A+pBMB251B2. (C) Strain BMB171/pBMB275A+pBMB251B3. (D) Strain BMB171/pBMB275A+pBMB251B5. (E) Strain BMB171/pBMB0617A+pBMB251B2. Magnification, ×1,000. Data not shown for pBMB251B4.

Firstly, pBMB251A (a fragment of pBMB28), was constructed by inserting the 35 kb fragment from pBMB251 into shuttle BAC vector pEMB0603, and then transferring this into BMB171. The subclones of the 35 kb fragment within pBMB275 (a fragment of pBMB26) were then transferred into strain BMB171/pBMB251A. The results demonstrated that a minimal fragment of 4 kb (pBMB0617), carrying an intact *cry26Aa* gene was sufficient for the SCA phenotype (Fig. 3A, Fig. 4A, Table 1). Following this, the subclones of the 35 kb fragment within pBMB251 (a fragment of pBMB28), were transferred into strain BMB171/pBMB275A. A 7 kb fragment (pBMB251B2), was confirmed as the minimal sufficient fragment for the SCA phenotype (Fig. 3B, Fig. 4B). Sequence analysis showed that this 7 kb fragment carried five putative genes (Table 1). Three partial sequences from this fragment could not recover the SCA phenotype in strain BMB171/pBMB275A, and separated crystals were formed after sporulation (Fig. 4C, 4D). The combination of the above described 4 kb and 7 kb fragments in strain BMB171 led to the SCA phenotype (Fig. 4E). This meant that the two fragments of 4 kb and 7 kb, from pBMB26 and pBMB28 respectively, contained the critical genetic information for the SCA phenotype. (Nucleotide sequences of two fragments have been deposited in GenBank under accession numbers DQ242519 and HQ695909).

**Table 1:** ORFs involved in the spore-crystal association (SCA) phenotype.

Gene	Position^a^	Size (aa)	Best BLAST match (source)	GenBank accession no.	% amino acids identity	reference
pBMB275						
*Cry26Aa*	7026–10520	1164	Cry26Aa [*Bacillus thuringiensis* subsp. *finitimus*]	ABB51652	100	[5]
pBMB251						
*orf1*	18621–19523	300	crystal protein NT40KD [*Bacillus thuringiensis* subsp *dakota*]	AAL26871	30	[11]
*orf2*	19625–20431	268	crystal protein NT32KD [*Bacillus thuringiensis* subsp *dakota*]	AAL26870	22	[11]
*orf3*	20796–22238	480	putative spore germination receptor [*Bacillus thuringiensis* subsp *israelensis*]	CAD30127	58	[14]
*orf4*	22231–23334	367	putative spore germination receptor [*Bacillus thuringiensis* subsp *israelensis*]	CAD30126	54	[14]
*orf5*	23331–24461	376	putative spore germination receptor [*Bacillus thuringiensis* subsp *israelensis*]	CAD30125	44	[14]

a The numbers correspond to the nucleotide coordinates of inserted fragments in pBMB275 or pBMB251.

DNA sequence analysis of the 7 kb fragment revealed that it contained five ORFs (Table 1). The genes *orf1* and *orf2* encoded the putative peptides of 300 and 268 amino acid residues with predicted molecular weights of 34,509 daltons and 30,872 daltons respectively. These two proteins showed low similarity with the crystal proteins NT40KD and NT32KD [11], exhibiting 30% and 22% sequence identity. Gene *orf1*, located 101 nucleotides upstream of *orf2* in the same orientation, was preceded by potential ribosome-binding sites. No putative promoter was found upstream of *orf2*. It is probable that the two genes are involved in a single operon. The genes *orf3*, *orf4*, and *orf5* were similar to the genes encoding the germination complex, and were particularly similar to the spore germination genes pBt086, pBt085, and pBt084, which are located on the plasmid pBtoxis. These had 58%, 54%, and 44% amino acid sequence identity with Orf3, Orf4, and Orf5 [12], [13]. This germination complex operon has been demonstrated to be a single operon by transcriptional analysis [14]. The three *orf*s shared sequence overlaps; *orf3*, for example, was shown to overlap with *orf4* by 8 bp, while *orf4* overlapped with *orf5* by 4 bp. This suggests that the three genes are organized within a single operon.

### Construction and analysis of mutant strains at the critical genes

**Figure 5:**
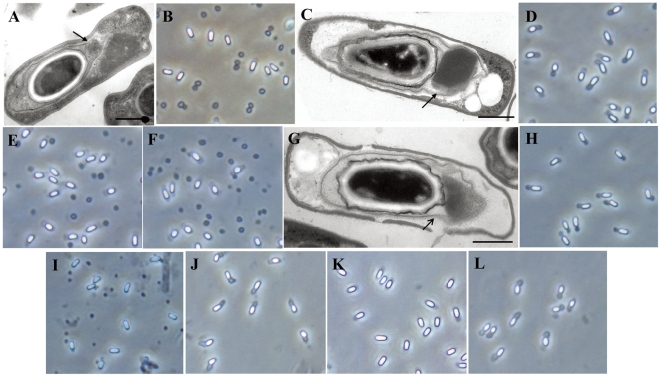
Micrographs of mutant strains of YBT-020 and their corresponding complementary strains. (A) and (B) Strain BMBJA (genes *orf1* and *orf2* disrupted). (C) and (D) Complementary strain BMBJA/pBMB251B5 (carrying *orf1* and *orf2*). (E) Complementary strain BMBJA/pBMB251B5A (carrying a frameshift at the *Acc*I site in *orf1*). (F) Complementary strain BMBJA/pBMB251B5B (carrying a frameshift at the *Eco*RI site in *orf2*). (G), (H) and (I) strain BMBJB (genes *orf3*, *orf4*, and *orf5* disrupted). (J) Complementary strain BMBJB/pBMB252B3 (containing *orf3*, *orf4*, and *orf5*). (K) Strain BMBJ1 (gene *cry26Aa* disrupted); (L) Complementary strain BMBJ1/pBMB0617 (carrying the gene *cry26Aa*). (A), (C) and (G) Electron micrographs of a thin sections during sporulation, arrowheads indicate exosporium. Scale bar = 0.5 µm. (B), (D), (E), (F), (K) and (L) after Growth for 48 h. (H) after growth for 36 h. (I) and (J) after growth for for 36 h, 100 h. Magnification, ×1,000.

To confirm that the six genes were involved in determining the SCA phenotype in strain YBT-020, three mutant strains were constructed and characterized. The operon containing *orf1* and *orf2* was disrupted to create mutant strain BMBJA. We observed that the crystals of this mutant were formed outside the exosporium during sporulation (Fig. 5A), and were separated from the spores after sporulation (Fig. 5B). In complementation experiments, the plasmid pBMB251B5 carrying *orf1* and *orf2* (2.9 kb, Fig. 3B), was capable of recovering the SCA phenotype in BMBJA (Fig. 5C, 5D).

To confirm that *orf1* and *orf2* are necessary to the SCA phenotype, a frameshift was introduced into these genes to generate plasmids pBMB251B5A and pBMB251B5B respectively (see Materials and Methods), These plasmids were transferred into strain BMBJA. No SCA phenotyope was observed in the transformants (Fig. 5E, 5F). The combination of the two frameshift mutant plasmids in strain BMBJA resulted in the SCA phenotype (data not shown). This indicated that both the products of *orf1* and *orf2* are indispensable for the SCA phenotype.

The operon containing *orf3*, *orf4*, and *orf5*, was disrupted, resulting in mutant strain BMBJB. Microscopic observation revealed that the crystals of this mutant formed inside the exosporium during sporulation (Fig. 5G) and the SCA phenotype remained after sporulation (Fig. 5H), However, the SCA phenotype in the mutant appeared impermanency compared with wild strain YBT-020. After growth for 100 h, the crystals were no longer attached to the spores (Fig. 5I). This observation suggested that the products of *orf3*, *orf4*, and *orf5* were only essential in maintaining the stability of the SCA phenotype, and were not involved in the formation of the SCA phenotype in strain YBT-020. In complementation experiments, the plasmid pBMB251B3 carrying intact *orf3*, *orf4*, and *orf5* (5.3 kb, Fig. 3B) was demonstrated to be capable of restoring the stability of the SCA phenotype in BMBJB (Fig. 5J).

Strain BMBJ1 was a *cry26Aa* gene disruption mutant from strain YBT-020. Microscopic observation revealed that BMBJ1 had lost the ability to form crystals (Fig. 5K). After the plasmid pBMB0617 harboring the *cry26Aa* gene was transferred into BMBJ1, the crystals were observed and the SCA phenotype was regained (Fig. 5L). This confirmed that the crystal protein gene, *cry26Aa*, was essential for the SCA phenotype. Through the characterization of mutant strains, we thus revealed that the six genes were indispensable to the formation of the SCA phenotype in strain YBT-020.

### Crystal proteins that are normally deposited outside the exosporium were not able to restore the SCA phenotype in BMBJ1

In a previous study [5], we found that the Cry1Ca protein could not be deposited inside the exosporium in strain YBT-020. To determine if the crystal proteins that were normally deposited outside the exosporium were able to replace Cry26Aa and restore the SCA phenotype in BMBJ1, several crystal protein genes, including *cry1Ac*, *cry2Aa*, *cry5Ba*, *cry6Aa*, *cry7Ba*, *cry51Aa*, and *cry55Aa*, were transferred into mutant strain BMBJ1 to detect the formation of the SCA phenotype. Microscopic observation revealed that only separated crystals were formed (data not shown). This suggested that the Cry26Aa protein was specific to the formation of the SCA phenotype in strain YBT-020.

## Discussion

Aronson [15] proposed two possible mechanisms for SCA phenotype formation. In the first, he suggested that the synthesis time of the crystal protein was synchronized with the formation of the exosporium. In a previous study [5], we demonstrated that crystal protein genes and their promoters were not able to cause crystal formation inside the exosporium. Thus, Aronson's second hypothesis seems reasonable. This was that the plasmid gene encodes a protein that binds the crystal protein to the inner surface of the exosporium. For determining the critical regions, we used a shuttle BAC library to carry out complementation testing and identified that five genes from pBMB28 and a crystal protein gene *cry26Aa* from pBMB26 were indispensable to the SCA phenotype in strain YBT-020. By screening shuttle BAC library, we have isolated several novel crystal protein genes [16], and a thuringiensin synthesis gene cluster [17]. Our results testify to the convenience of this method in searching for unknown functional genes or gene clusters. This is the first report of the isolation of genes determining the SCA phenotype.

When other types of crystal protein genes were transferred into strain BMBJ1, only separated crystals were observed under phase microscope. This suggested that the formation of the SCA involved recognition of the specific amino acid sequences of the Cry26Aa protein. We also isolated the gene *cry28Aa* from strain YBT-020 as well, and by screening the library of YBT-020, we isolated a fragment of pBMB28 carrying the *cry28Aa* gene. The combination of this fragment and plasmid pBMB0617 harboring the *cry26Aa* gene in strain BMB171 was able to prolong the stability of separated inclusion bodies (unpublished). We speculated that the co-existence of proteins Cry26Aa and Cry28Aa provided a way of preventing the crystal protein from degradation. The genes *orf1* and *orf2* were found to determine the deposition of crystal proteins inside the exosporium. The genes *orf3*, *orf4* and *orf5* were found to be necessary for the stability of the SCA phenotype. This implied that the formation of the SCA phenotype was involved in protein interaction and was closely linked with spore differentiation and development. We have revealed the genes which were indispensable to the SCA phenotype. However, the functions of the proteins encoded by these genes remain unclear and need investigation.

The exosporium is a prominent structure with a paracrystalline basal layer and an external hair-like nap [18]. It is composed of at least 20 proteins and glycoproteins [19]. Exosporium assembly is a non-uniform process, and exosporium formation begins with the synthesis of a cap substructure [20]. How do proteins, which are essential to the formation of SCA phenotype, carry out their function during the process of SCA? We propose the following explanatory mechanism to explain it. The Orf1 and Orf2 proteins play a role as bridges between spores and crystals. We infer that these two proteins are located on the spore coat, and interact with crystal proteins and other spore proteins (such as the products of the *orf3*, *orf4*, and *orf5* operon). They would therefore bind crystal proteins to the inner surfaces of the exosporium cap. The inclusion assembly could then be confined to such a site in order to ensure a location within the exosporium. This needs detailed investigation in future.

Untill now, many strains with SCA phenotype have been isolated, and the crystal proteins enclosed within the exosporium are distinct among subspecies. Four morphological types of the SCA phenotype and four different crystal protein profiles were isolated from citrus orchards in Spain [8]. Genome sequence analysis of *B. thuringiensis* strain C15 (another strain with the SCA phenotype maintained in our lab), showed that the genes involved in the formation of the SCA phenotype in YBT-020 could not be found (data not shown). This indicates that the genes controlling the formation of the SCA phenotype differ among subspecies.

## Materials and Methods

### Bacterial strains, plasmids, growth conditions and DNA manipulations

The bacterial strains and plasmids involved in this study are listed in Table 2. Conditions and media used for growing and maintaining different strains of *E. coli* and *B. thuringiensis* have been described previously [16]. Chloromycetin, tetracycline, erythromycin, kanamycin, ampicilin, and rifampin were supplemented at the final concentrations of 5 or 25, 10, 25, 50, 100, 100 µgmL^−1^ when needed, respectively. Plasmids were extracted from *B. thuringiensis* following the procedure described by Andrup *et al.* [21]. All regular DNA manipulations were carried out following standard methods [22]. *E. coli*-*B. thuringiensis* shuttle vectors, pEMB0557 and pEMB0603, were used to clone large DNA fragments. The two vectors were constructed from BAC vector pBeloBAC11 by adding erythromycin and kanamycin resistance genes, as the selectable marker in *B. thuringiensis*, and *B. thuringiensis*-originated plasmid replication origins *ori60* and *ori44* respectively. The 2.3-kb *ori60* and 2.25-kb *ori44* replication origins were amplified from large plasmids in *B. thuringiensis* strain YBT-1520 [23], and show 99% and 100% sequence identity with that of the 91 kb and 66 kb plasmids in *B. thuringiensis* subsp. *kurstaki* HD-263 [24]. *B thuringiensis* transformation was conducted as described previously [25].

**Table 2:** Bacterial strains and plasmids.

Strain or plasmid	Description^a^	Source or reference
*Bacillus thuringiensis*		
YBT-020	*B. thuringiensis* subsp. *finitimus*, harboring pBMB26 and pBMB28	[5]
BMB171	Acrystalliferous mutant of *B thuringiensis* subsp. *kurstaki*	[33]
BMB171R1	BMB171R containing pBMB0617.	This work
BMB1151	YBT-020 derivative, containing pBMB28 but cured of pBMB26	[9]
BMBJ1	YBT-020 derivative, with a chloromycetin insertion at *cry26Aa* gene	This work
BMBJA	YBT-020 derivative, with a chloromycetin insertion replacing the DNA fragment covering *orf1* and *orf2*	This work
BMBJB	YBT-020 derivative, with a chloromycetin insertion replacing the DNA fragment covering *orf3*, *orf4* and *orf5*	This work
*Escherichia coli*		
DH5α	F- *ϕ80lacZΔM15 Δ(lacZYA-argF) U169 deoR recA1 endA1 hsdR17 (rk-,mk+) phoA supE44 λ- thi-1 gyrA96 relA1*	
EPI300	F^−^ *mcrA Δ(mrr-hsdRMS-mcrBC) Φ80dlacZΔM15 ΔlacX74 recA1 endA1 araD139 Δ(ara, leu)7697 galU galK λ^−^ rpsL (Str^R^) nupG trfA tonA*	Epicentre
Plasmids		
pHT304	*E. coli* and *B. thuringiensis* shuttle vector; Amp^r^, Erm^r^	[34]
pDG1514	*E .coli* vector. Amp^r^, Tet^r^	[35]
pEG491	*E. coli* and *B. thuringiensis* shuttle vector; Amp^r^, Cm^r^	[27]
pHT304Ts	Derivative of pHT304, containing temp-sensitive replicon, 6.8 kb	This work
pEMB0557	*E. coli* and *B. thuringiensis* shuttle BAC vector; Cm^r^, Erm^r^	[26]
pEMB0603	*E. coli* and *B. thuringiensis* shuttle BAC vector; Cm^r^, Kan^r^	unpublished
pBMB26	188 kb endogenous plasmid harboring *cry26Aa* in strain YBT-020	[5]
pBMB28	139 kb endogenous plasmid harboring *cry28Aa* in strain YBT-020	[5]
pBMB26-Cm^R^	Derivative of pBMB26, with a chloromycetin insertion at *cry26Aa* gene	This work
pBMB275	pEMB0557 containing a 35 kb fragment of pBMB26	This work
pBMB275A	pEMB0603 containing a 35 kb fragment of pBMB26	This work
pBMB0617	pHT304 containing *cry26Aa*	[5]
pBMB0617A	pEMB0603 containing *cry26Aa*	This work
pBMB290	pEMB0557 containing 16.7 kb *Bam*HI fragment of pBMB275	This work
pBMB291	pEMB0557 containing 13.7 kb *Hin*dIII fragment of pBMB275	This work
pBMB292	pHT304 containing 6.3 kb *Bam*HI/*Sal*I fragment of pBMB275	This work
pBMB251	pEMB0557 containing a 35 kb fragment of p28	This work
pBMB251A	pEMB0603 containing a 35 kb fragment of p28	This work
pBMB251B1	pHT304 containing 12.5 kb *Bam*HI fragment of pBMB251	This work
pBMB251B2	pHT304 containing 7 kb *Sca*I/*Hin*dIII fragment of pBMB251	This work
pBMB251B3	pHT304 containing 5.3 kb *Pst*I/*Hin*dIII fragment of pBMB251	This work
pBMB251B4	pHT304 containing 1.7 kb *Sca*I/*Eco*RI fragment of pBMB251	This work
pBMB251B5	pHT304 containing 2.9 kb *Sca*I/*Hin*dIII fragment of pBMB251	This work
pBMB251B5A	pBMB251B5 derivative carrying frameshift mutation at *Acc*I site of *orf1*	This work
pBMB251B5B	pBMB251B5 derivative carrying frameshift mutation at *Eco*RI site of *orf2*	This work

a Amp^r^, ampicillin resistance; Cm^r^, Chloromycetin resistance. Erm^r^, erythromycin resistance; Kan^r^, kanamycin resistance; Tet^r^, Tetracycline resistance.

### Construction of shuttle BAC library and screening of clones covering the full length of plasmids pBMB26 and pBMB28

Shuttle vector pEMB0557 was used to construct a genomic BAC library of *B. thuringiensis* strain YBT-020. Construction of library was performed following the method described by Liu et al [26], with slight modifications. Genomic DNA embedded in agarose plugs was partially digested with *Hin*dIII and separated by pulse field gel electrophoresis (PFEG). 30–50 kb fragments of genomic DNA were recovered by electroelution (Bio-Rad) and were ligated with *Hin*dIII dephosphorylated vector pEMB0557 to generate a genomic library. The fragments which covered the full length of pBMB26 and pBMB28 were screened from library of strain YBT-020.

### Construction of recombinant plasmids

#### (i). Large fragment complementation testing

Two fragments, which were seclected from the shuttle BAC library and were able to recover the SCA phenotype in BMB171, were named as pBMB275 and pBMB251. And then, they were sub-cloned into pHT304 for sequencing by a primer walking strategy. The two fragments were digested with *Not*I and inserted into another vector pEMB0603 to generate pBMB275A and pBMB251A.

#### (ii). Minimizing the regions that determine the SCA phenotype

Deletion derivatives of pBMB275 containing either a *Bam*HI fragment of 16.7 kb or a *Hin*dIII fragment of 13.7 kb were designated pBMB290 and pBMB291 respectively. A 6.3 kb *Nde*I fragment from pBMB291 was first inserted into pDG1514, then digested with *Bam*HI and *Sal*I, and inserted into pHT304 to generate pBMB292. A fragment of 4 kb was amplified from pBMB275 using the pair of primers cry26-F and cry26-R (Table 3). This fragment was cloned into pEMB0603 between *Bam*HI and *Hin*dIII to generate pBMB0617A. Deletion derivatives of pBMB251, which containing either a 12.5 kb *Bam*HI fragment, a 7 kb *Sca*I-*Hin*dIII fragment, a 5.3 kb *Pst*I-*Hin*dIII fragment, or a 1.7 kb *Sca*I-*Eco*RI fragment, were designated pBMB251B1, pBMB251B2, pBMB251B3, and pBMB251B4, respectively. A fragment of 2.9 kb was amplified from pBMB251 using the the pair of primers 251B1 and 251B2 (Table 3), and cloned into pMD18-T simple vector to generate pEMB251B5. After that, it was digested with *Sca*I and *Hin*dIII and then cloned into pHT304 between *Sma*I and *Hin*dIII, to generate pBMB251B5.

**Table 3:** Primers used for PCR.

Primer	Sequence	Construct(s)	Use
cry26-F	CCCTGGATCCGGAAATAAACGAACCTTCA	pBMB0617A	Heterologous expression
cry26-R	CGCGAAGCTTTGGTGATGTTAAGCCCATAT		
251B1	CCCGAGTACTTAAATAATAGCCT	pBMB251B5	Heterologous expression
251B2	CCCAAAGCTTATAGGTACGATACTAC		
251B4	CCAGAATTCATGCCCATCAACT	pEMBJA	Gene disruption
251B5	CGGTAGAATTCGAGTTGTACTTTCT		
251B6	CAGGATCCGTATCGTACCTATA	pEMBJB	Gene disruption
251B7	CGCAAGCTTACCATCAAATGC		
251B8	AAGGAATTCAACT GGGGT CCTTC	pEMBJB	Gene disruption
251B9	TACGAATTCGCGGCTCATAACT		
cry26A	AATGGATCCAAACGAACCTTCATTCA	pEMBJ1	Gene disruption
cry26B	GGCTGGATCCTTAACTTTGTATTTCC		
CM1	AAAGAATTCTTCGCTACGCTCAAATC	pEMBJ1	Gene disruption
CM2	TCAGAATTCTTCACCGTCATCACCGA		

#### (iii). Construction of frame-shift mutant plasmids

To assess the role of Orf1 and Orf2 protein, two plasmids pBMB251B5A and pBMB251B5B were constructed as follows: Briefly, frame shift mutation was introduced in the pEMB251B5 by *Acc*I and *Eco*RI cleaving respectively, filling-in of the recessed ends with Klenow enzyme, and then blunt-end self-ligated. This was then digested with *Sca*I and *Hin*dIII and cloned into pHT304 between *Sma*I and *Hin*dIII, to generate pBMB251B5A and pBMB251B5B. The DNA sequencing confirmed the effectiveness of the mutation by introduction of stop codons generating truncated *orf1* and *orf2*.

### Construction of gene disruption mutants of strain YBT-020

To construct a temperature-sensitive shuttle vector pHT304Ts, an *Eco*RV fragment of 2.1 kb, carrying the replication protein gene from the *E.coli-B. thuringiensis* shuttle vector pHT304 was replaced by a *Hpa*I fragment containing the temperature-sensitive replication origin from pEG491 [27].

To verify the necessary of the genes for SCA in strain YBT-020, three gene disruption strains were constructed via homologous recombination. The first was the *cry26Aa* disruption strain BMBJ1. Using primers cry26A and cry26B (Table 3), a fragment of 2 kb carrying the partial region of *cry26Aa* was amplified by PCR, cloned into pMD18-T simple vector, and then digested with *Eco*RI. A chloramphenicol resistance cassette, derived from pAD123, was amplified with primers CM1 and CM2 (Table 3), and cloned into the *Eco*RI site, yielding pEMBJ1. The *Hin*dIII fragment was inserted into pHT304Ts to generate pBMBJ1.

To generate the *orf1* and *orf2* deletion mutant strain BMBJA, a fragment of 3.8 kb was amplified by reverse PCR with primers 251B4 and 251B5 (Table 3) using pEMB251B5 as template, and then digested with *Eco*RI. A chloramphenicol resistance gene was amplified and cloned into the *Eco*RI site to give rise of pEMBJA. The plasmid was digested by *Sca*I and *Hind*III and inserted into pHT304Ts between *Sma*I and *Hin*dIII to generate pBMBJA.

To obtain the operon deletion mutant strain for *orf3*, *orf4*, and *orf5*, a PCR fragment of 3.8 kb was amplified with primers 251B6 and 251B7 (Table 3) using pBMB251 as template, and inserted into pMD18-T simple vector to generate pEMBJB. Using the plasmid as template, primers 251B8 and 251B9 (Table 3) were used to amplify a 4 kb fragment by reverse PCR. This was then digested with *Eco*RI. A chloramphenicol resistance gene was amplified and cloned into the *Eco*RI site, yielding pEMBJB1 which was then digested by *Bam*HI and *Hind*III and inserted into pHT304Ts to generate pBMBJB.

Mutants were selected following the method described by Fang *et al.* [28]. Briefly, the plasmids were transferred into strain YBT-020, and the transformants were cultivated in LB medium with 2.5 µg/mL chloramphenicol for 8 h. Then the transformants were incubated at 42°C for 4 days to eliminate unintegrated temperature-sensitive plasmids. The expected mutant strains, which were resistant to Chloramphenicol and meanwhile sensitive to erythromycin colonies, were harvested and confirmed by PCR using appropriate primers and sequencing.

### Conjugation experiments

Plasmid conjugation transfers were conducted following the protocols described by Andrup *et al.* [29] with slight modifications. In short, the overnight cultures of donor and recipient strains were incubated separately at 28°C in LB medium with appropriate antibiotics. Equal quantities of donor and recipient cells (250 ml per OD_600_ unit) in logarithmic growth were mixed and shaken in 5 ml prewarmed LB medium at 28°C with moderate shaking (80 rpm). After 8 h, appropriate dilutions were plated onto appropriate selective medium to determine the number of transconjugants Controls of donors and recipients grown separately were also tested.

### Microscopic observation

For phase contrast microscopy, all *B. thuringiensis* were sporulated at 28°C and 220 rpm in a liquid medium (ICPM medium) containing 0.6% peptone, 0.5% glucose, 0.1% CaCO_3_, 0.05% MgSO_4_, and 0.05% KH_2_PO_4_ (pH 7.0) until almost all mother cell lysis. Spores and crystals were collected by centrifugation and washed three times with a solution containing 1 mol of NaCl per liter and then three times with water. The mixture of spores and crystals was then resuspended in water [30]. By phase-contrast microscopy, spores appear as phase-bright, and crystals appear as phase-dark [31]. Each sample was chosen five fields to observe morphology. Transmission electron microscopy was performed following the method described by Bailey-Smith *et al.* [32]. Sections were examined under a FEI Tecnai G^2^ 20 TWIN transmission electron microscope at an accelerating voltage of 200 kV.
